# Intrauterine Bakri balloon tamponade plus cervical cerclage for the prevention and treatment of postpartum haemorrhage in late pregnancy complicated with acute aortic dissection: Case series

**DOI:** 10.1515/med-2021-0392

**Published:** 2021-11-27

**Authors:** Mei Peng, Ling Yu, Yali Deng, Wen Zhong, Yanting Nie, Wen Deng, Jian Huang, Yiling Ding

**Affiliations:** Department of Obstetrics, The second Xiangya Hospital, Central South University, Changsha 410011, People’s Republic of China

**Keywords:** pregnancy, Stanford type A aortic dissection, postpartum bleeding, Bakri balloon, intrauterine tamponade, cervical cerclage

## Abstract

In this study, a treatment method was assessed for the prevention and treatment of postpartum bleeding after combined surgery in patients having late pregnancy with the complication of acute Stanford type A aortic dissection. The clinical records of ten patients receiving treatment at the Second Xiangya Hospital of Central South University between March 2012 and March 2021 were retrospectively analysed. All patients were diagnosed with acute Stanford type A aortic dissection according to computed tomography angiography of the thoracic and abdominal aorta. Aortic valve function was assessed using two-dimensional echocardiography. All patients experienced uterine-incision delivery under systemic anaesthesia. During the operation, intrauterine Bakri balloon tamponade and cervical cerclage were performed. Postpartum bleeding was effectively controlled for all patients. The extracorporeal circulation time was 230–295 min, the postpartum 24 h bleeding volume was 500–870 mL, the volume of physiological saline injected into the balloon was 290–515 mL, and the intrauterine balloon compression time was 28–51 h. No postpartum bleeding occurred. A 42-days follow-up showed no late postpartum bleeding, poor uterine incision healing, or puerperal infection, and no uterine removal was performed. Intrauterine Bakri balloon tamponade plus cervical cerclage can effectively prevent intra- and postoperative postpartum bleeding in pregnant patients with aortic dissection.

## 1 Introduction

Acute Stanford type A aortic dissection (referred to as acute type A aortic dissection hereafter) is a life-threatening, critical clinical condition. In the absence of timely diagnosis and treatment, approximately 50% of the patients can die within 1 week [1]. Pregnancy is an independent risk factor for aortic dissection [2]. During pregnancy, the cardiac and vascular volume load may increase due to placental circulation, uterine enlargement, endocrine changes, and increased circulation blood volume, which is likely to cause expansion, dissection, or even rupture of the aorta. Statistical results show that 50% cases of aortic dissection occur among pregnant women under the age of 40 years [3]. Data from foreign heart centres show that pregnancy significantly increases the risk of aortic dissection [4]. Doctors have insufficient experience in preventing and treating postpartum haemorrhage after combined surgery for pregnancy complicated with aortic dissection, particularly late pregnancy with acute type A aortic dissection. Whole-body heparinisation is needed to set up extracorporeal circulation during combined surgery, and the use of anticoagulant/antiplatelet drugs is likely to cause atonic haemorrhage from the caesarean section wound, which considerably increases the risk of postpartum haemorrhage, which in turn increases the hysterectomy rate by 20–30% or more [5]. Consequently, hysterectomy during caesarean section was proposed to prevent postpartum haemorrhage and hysterectomy in a single-centre study [6]. However, uterine removal negatively affects ovarian hormone secretion and accelerates ovarian failure, which in turn affects the endocrine state of the entire body [7]. Therefore, the view of our centre is that considerable caution should be exercised when deciding whether to perform a hysterectomy during caesarean section.

Here we retrospectively analysed the clinical data of ten pregnant patients with acute type A aortic dissection who received treatment at the Second Xiangya Hospital of Central South University between March 2012 and March 2021. Effective prevention and treatment methods for postpartum haemorrhage were explored for this population. This hospital serves as the emergency maternal treatment centre of Hunan Province. Pregnant patients with acute aortic dissection can receive timely treatment via the green channel, and the time span from patient admission to diagnosis and delivery is 2–24 h.

## 2 Clinical data and methods

### 2.1 General data

Clinical data were collected for ten pregnant patients with acute type A aortic dissection who received treatment at the Second Xiangya Hospital of Central South University between March 2012 and March 2021. The age of patients ranged from 22 to 43 years. The number of gestational weeks of the patients at the time of disease occurrence ranged from 28^+3^ w to 37^+4^ w, and the patient weight ranged from 61 to 71 kg, with a body mass index (BMI) of 21–26. All patients were diagnosed with acute Stanford type A aortic dissection according to computed tomography (CT) angiography of the thoracic and abdominal aorta, and aortic valve function was assessed using two-dimensional echocardiography. Among the patients, 4 (40%) had Marfan’s syndrome, among which 2 presented with aortic valve insufficiency; 3 (30%) had hypertension, among which 2 (20%) presented with diabetes, of which 1 case was complicated with antiphospholipid syndrome, and the foetus died in the uterus on admission; and 1 (10%) had no fundamental disease. The primary symptoms of these patients mainly included sudden poststernal pain, with upper abdominal and subxiphoid pain, and some patients also presented with dyspnoea, vomiting, and blurred vision (Table 1).

**Table 1:** General data of ten pregnant patients with acute type A aortic dissection

Patient no.	Age	Pregnancy and childbirth times	Gestational weeks	Fundamental disease	D-dimer (μg/L)	BMI	Diagnostic method	Primary symptom
1	43	G2P1A1	28^+5^	Diabetes + antiphospholipid syndrome	4.53	24	CTA + TEE	Poststernal pain + upper abdominal pain + subxiphoid pain, complicated with dyspnoea, vomiting, and amaurosis
2	39	G5P1A3	31	Marfan’s syndrome	2.58	23	CTA + TTE	Poststernal pain
3	26	G1P0A0	32^+4^	Marfan’s syndrome	3.56	25	CTA + TTE	Chest pain + chest distress
4	29	G3P1A1	34	Marfan’s syndrome	3.49	23	CTA + TTE	Upper abdominal pain
5	30	G2P1A0	32^+1^	Chronic hypertension	4.23	22	CTA	Chest and back pain, vomiting, and blurred vision
6	32	G4P1A2	36^+3^	Pregnancy-induced hypertension	3.02	26	CTA + TEE	Chest and subxiphoid pain
7	35	G2P0A1	37^+1^	Pregnancy-induced hypertension	2.49	24	CTA + TEE	Middle and upper abdominal pain + back pain
8	36	G3P1A1	37^+4^	None	3.06	21	CTA	Chest and back pain
9	38	G4P1A3	33	Pregnancy-induced hypertension	4.21	22	CTA + TTE	Chest pain + dyspnoea
10	22	G4P3A0	33^+1^	Marfan’s syndrome	3.12	22	CTA + TTE	Persistent tearing pain in chest and back

All patients provided informed consent for publication of the cases.

### 2.2 Diagnosis

According to the Expert Consensus on Diagnosis and Treatment of Aortic Dissection of China, imaging diagnosis was performed using computed tomography angiography (CTA)/magnetic resonance imaging, transthoracic echocardiography (TTE), or transesophageal echocardiography (TEE). The ten cases of aortic dissection were typed according to Stanford criteria [8].

### 2.3 Treatment

An emergency green channel was immediately set up for the patients after hospitalisation. Experts from the obstetrics, neonatology, critical care, cardiac surgery, vascular surgery, interventional operation, anaesthesiology, extracorporeal circulation, and blood transfusion departments were organised into a multi-disciplinary treatment emergency team that performed combined surgery on the patients.

#### 2.3.1 Preoperative treatment

Emergency surgery is required for patients with acute type A aortic dissection. Preoperative preparation was completed as early as possible. The patient’s blood pressure was strictly controlled, and the heart rate was reduced to an appropriate level. Particular attention was given to pain, which was alleviated with intramuscular sauteralgyl injection. Before aortic dissection surgery, the patient’s CT image was carefully read and discussed. CT reconstruction was used to determine the proximal and distal positions of the tear, and a preoperative strategy was formulated.

#### 2.3.2 Operation

**Figure 1:**
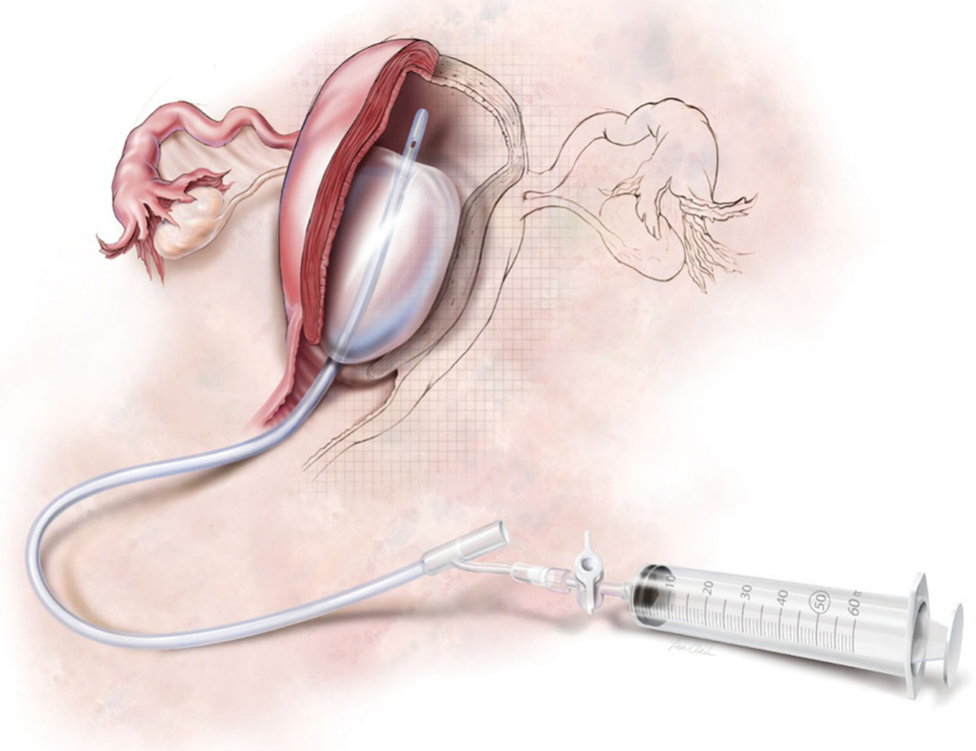
Bakri balloon.

Caesarean section was performed through an abdominal longitudinal incision in the lower segment of the uterus by experienced senior surgeons (associate professors or professors). The operation was completed as quickly as possible to leave sufficient time for vascular surgery. During the operation, complete haemostasis and high-quality suturing of the wound were required to reduce the chance of postoperative bleeding and infection. Bakri balloon (Figure 1) tamponade and transvaginal cervical cerclage were performed by obstetricians according to the procedures described in refs. [9] and [10], respectively. Then, vascular surgery was performed by cardiothoracic surgeons.

Forty-eight hours after the operation, the fluid in the uterine balloon was slowly drawn out, and the balloon was pulled out when no bleeding was observed. Then, the cervical cerclage suture was removed.

#### 2.3.3 Perioperative treatment

Broad-spectrum antibiotics (normally second-generation antibiotics, such as cefoxitin sodium) were routinely administered to the patients during the perioperative period. In addition, cervical secretions were preserved for a drug sensitivity experiment. In case of condition changes, antibiotics were adjusted in a timely manner.

### 2.4 Postoperative observation and follow-up

#### 2.4.1 Observation

The blood volumes from the uterine balloon drainage tube and packing sheet were closely observed and recorded. Blood loss during extracorporeal circulation was recorded at least every two hours and blood loss after vascular surgery was recorded at least every four hours. The postpartum 24 h blood loss was calculated. It was carefully observed whether the drainage tube was twisted, compressed, or displaced and if there was blood on the abdominal wound dressing.

The volume of physiological saline injected into the intrauterine balloon was recorded.

#### 2.4.2 Follow-up

After discharge from the hospital, all patients were followed up by the outpatient system of our centre. The patients were asked to visit the Department of Obstetrics on the 7th, 14th, and 42nd day after childbirth.

### 2.5 Ethical statements

The procedures of this study were approved by the Ethics Committee of the Second Xiangya Hospital of Central South University.

## 3 Results

After a multidisciplinary consultation, all the ten patients were subjected to combined surgery. Under systemic anaesthesia, uterine-incision delivery + intrauterine Bakri balloon tamponade (transvaginal) were performed, followed by Bentall surgery. Four patients received extra-total-aortic-arch replacement and “descending-aortic-stent”-elephant-trunk implantation, three patients received extra-coronary-artery-bypass grafting, and two received extra-vertebral-artery transplantation. Three patients underwent transvaginal-balloon resetting because relaxation of the uterine orifice caused balloon detachment during the operation. All patients survived; intrauterine stillbirth occurred in one patient, and nine newborns survived with satisfactory prognoses. Haemorrhage was effectively controlled for all patients, with an extracorporeal circulation time of 230–295 min, a 24 h vaginal blood loss after childbirth of 500–870 mL, 290–515 mL of physiological saline injected into the balloon, and 28–51 h of intrauterine-balloon compression. The intrauterine balloon was extracted, and the cervical cerclage suture was removed (Table 2). The ICU stays of the patients ranged from 3 to 5 days, and the general ward stays ranged from 15 to 18 days. The patients were followed up to 42 days after childbirth. No late postpartum haemorrhage, poor uterine incision healing, or puerperal infection occurred. No uterine removal was performed.

**Table 2:** Blood loss and the volume of physiological saline injected into the intrauterine balloon within 24 h after caesarean section

Index	No. 1	No. 2	No. 3	No. 4	No. 5	No. 6	No. 7	No. 8	No. 9	No. 10
Extracorporeal circulation time (min)	232	230	271	248	257	226	241	295	274	261
Blood loss during caesarean section (within 1 h of operation)/physiological saline volume injected into the balloon (mL)	150/170	200/200	250/220	180/160	200/200	300/200	200/200	280/230	200/180	200/200
Blood loss within 1–2 h of delivery/physiological saline volume newly injected into the balloon (mL)	50/20	40/25	60/35	50/30	60/20	40/20	70/30	80/50	70/30	50/20
Blood loss within 2–4 h of delivery/physiological saline volume newly injected into the balloon (mL)	60/25	50/20	60/35	50/20	40/20	50/30	60/30	100/60	80/40	50/20
Blood loss within 4–6 h of delivery/physiological saline volume newly injected into the balloon (mL)	80/35	70/40	80/50	100/60	100/60	120/80	90/45	180/65	100/60	60/25
Blood loss within 6–8 h of delivery/physiological saline volume newly injected into the balloon (mL)	50/20	50/10	50/30	50/20	50/10	50/40	50/30	50/30	50/30	30/15
Blood loss within 8–10 h of delivery/physiological saline volume newly injected into the balloon (mL)	40/10	50/5	50/30	50/20	50/5	50/40	50/25	50/25	50/25	40/15
Blood loss within 10–12 h of delivery/physiological saline volume newly injected into the balloon (mL)	30/5	40/5	40/25	40/15	40/5	40/30	40/20	40/20	40/20	30/15
Blood loss within 12–14 h of delivery/physiological saline volume newly injected into the balloon (mL)	20/5	30/5	30/15	30/15	30/5	30/25	30/15	30/15	30/15	20/10
Blood loss within 14–16 h of delivery/physiological saline volume newly injected into the balloon (mL)	10/0	30/5	30/15	30/10	30/5	30/25	30/10	30/10	30/15	20/10
Blood loss within 16–20 h of delivery/physiological saline volume newly injected into the balloon (mL)	10/0	20/0	20/10	20/5	10/0	20/10	20/10	20/10	20/10	10/0
Blood loss within 20–24 h of delivery/physiological saline volume newly injected into the balloon (mL)	0/0	< 10/0	10/5	< 10/0	< 10/0	< 10/0	< 10/0	< 10/0	< 10/0	10/0
Total blood loss within 24 h of delivery/total physiological saline volume in the balloon (mL)	500/290	590/315	680/470	600/350	610/330	740/500	650/415	870/515	630/425	520/330
Balloon placement time (h)	28	31	32	40	42	47	40	51	48	38

## 4 Discussion

The prevalence of pregnancy complicated with aortic dissection is approximately 0.4–0.5/100,000 [11]. The incidence of aortic dissection complication in pregnancy has exhibited an increasing trend in the past few years. In our hospital, a total of 46 patients received treatment for aortic dissection during pregnancy in the past 9 years (from 2012 to 2021). The incidence rate of aortic dissection was approximately 0.17% for pregnant women in the third trimester. Aortic dissection has been reported to lead to a mortality as high as 60% and therefore, remains the primary cause of pregnancy complicated with cardiovascular disease-related deaths [12]. Stanford type A aortic dissection is the most frequent type of aortic dissection and is associated with an extremely high mortality, where approximately 21% of the patients die out of hospital [13]. During pregnancy, maternal haemodynamics and haematology change significantly, as manifested by an accelerated heart rate, an increase in cardiac output, increased left ventricular thickness, etc. During late gestation, the plasma volume increases by 45%, and the red blood cell mass increases by 20%. Furthermore, the expanded uterus compresses the abdominal part of the aorta and the iliac artery, which leads to gestational hypertension [14,15,16]. Increased oestrogen and progesterone levels during pregnancy can cause noticeable changes in the structure of the aortic wall, including elastic layer damage, proteoglycan reduction, and hypertrophy and hyperplasia of the vascular smooth muscle [17]. These changes are most noticeable during late pregnancy and the early postpartum stage and are more significant in pregnant women with vascular disease, such as hypertension, diabetes, and potential aortic disease (e.g. Marfan’s syndrome), increasing the risk of aortic dissection [18].

Pregnancy complicated with aortic dissection is virulent. Postpartum bleeding after combined surgery is hard to control, and the conventional treatment is likely to cause postpartum haemorrhage, leading to uterine removal or even posing a life threat. During vascular surgery after caesarean section for pregnant patients with acute aortic dissection, the extracorporeal circulation has to be completely heparinised, and the administration of postoperative anticoagulant/antiplatelet drugs causes coagulation dysfunction and increases the risk of postoperative haemorrhage. Postpartum haemorrhage induced by these postoperative drugs differs from that caused by other factors, such as uterine atony, placental factors, and soft birth canal injury, because coagulation dysfunction is a prerequisite for performing extracorporeal circulation. The first treatment choice for patients with postpartum haemorrhage is the infusion of fresh whole blood, platelets, fibrinogen or prothrombin complex, coagulation factor, etc., and is not suitable for those undergoing extracorporeal circulation. Uterotonics can also reduce bleeding by strengthening uterine contraction. However, local administration could not be performed on the patients in this study after extracorporeal circulation was started because the abdomen had been closed; peripheral administration was not an option because the drug concentration would have been considerably reduced due to drug dilution and membrane adsorption caused by extracorporeal circulation. Under such conditions, intrauterine balloon compression can achieve a satisfactory haemostatic effect on postpartum haemorrhage [19].

Intrauterine Bakri balloon tamponade has been extensively and effectively applied as a haemostatic measure in clinical practice in recent years. The balloon is designed according to the physiological features of the postpartum uterine cavity. After intrauterine tamponade, saline infusion is used to expand the balloon by increasing the intrauterine pressure to compress uterine spiral arteries, thereby achieving haemostasis. Although Bakri balloons are easy to pack and have satisfactory haemostatic effects, the balloon tamponade has a haemostasis failure rate of 2.2–20.0% due to inappropriate application, improper timing, and poor tamponade skills [20], with balloon shedding as the main cause of haemostasis failure. In this study, all patients received intrauterine Bakri balloon tamponade and cervical cerclage, which effectively controlled haemorrhage during and after the combined surgery. During and after vascular surgery, bleeding from the uterine wound can be well-controlled by regulating the liquid volume in the balloon. This adjustment is easy to perform and has satisfactory repeatability, which considerably reduces the occurrence of bleeding and uterine removal. Cervical cerclage fixes the compression position of the Bakri balloon, which effectively prevents balloon shedding. This technique is easy to operate and does not require special consumable materials. The technique does not increase the economic burden on patients, while producing an effective haemostatic effect. Bakri balloon tamponade combined with cervical cerclage has a noticeably better haemostatic effect than intrauterine gauze packing combined with uterine binding. However, if bleeding reoccurs during vascular surgery, neither of these two combination methods should be performed repeatedly. Even worse, both methods are likely to increase the risk of infection and uterine adhesions. In this study, all patients received intrauterine balloon tamponade combined with cervical cerclage. No balloon shedding occurred. The patients were followed up to 42 days after childbirth, and no late postpartum bleeding or puerperal infection occurred.

Pregnancy complicated with acute aortic dissection has a rapid onset and a high mortality, making early identification particularly important. Pain is the most frequent symptom of acute aortic dissection [21]. Although satisfactory pregnancy outcomes of patients with a history of acute aortic dissection have been reported, there is insufficient evidence for safe pregnancies of patients with fertility needs who have a history of acute aortic dissection [22]. Therefore, for patients with fertility needs and a high risk of aortic dissection, the vascular state should be adequately assessed prior to pregnancy, risks should be fully communicated to the patient, and fundamental diseases should be strictly controlled. Patients should select general hospitals with diagnosis and treatment experience for pregnancy examination and delivery.

A hysterectomy can prevent uncontrollable bleeding from uterine wounds caused by systemic heparinisation of extracorporeal circulation but may affect patients’ psychosomatic health [6,7]. Therefore, to prevent and treat postpartum haemorrhage in pregnancy complicated with acute aortic dissection at our centre, intrauterine balloon compression was used combined with cervical cerclage to prevent balloon shedding, with a satisfactory therapeutic effect. For those who do not need to continue pregnancy, bilateral tubal ligation is performed. If the haemostatic effect of balloon compression is not satisfactory, bilateral uterine artery embolisation can be considered, with hysterectomy as the last treatment choice. Additionally, in early puerperium, attention should be given to postpartum haemorrhage caused by coagulation dysfunction due to the large consumption of coagulation factors, fibrinogen, and platelets during the formation of dissection, as well as the destruction of a large quantity of coagulation factors during extracorporeal circulation [23,24]. Timely component blood transfusion should be performed after operation. Moreover, due to the continuous packing of the intrauterine balloon, surgeons also need to remain alert to intrauterine infection. Therefore, prophylactic antibiotics should be administered after operation. Timely balloon removal is necessary to ensure the haemostatic effect of intrauterine balloon compression (intrauterine balloon compression generally does not exceed 48 h).

In conclusion, intrauterine Bakri balloon tamponade combined with cervical cerclage can achieve satisfactory results for the prevention and treatment of postpartum haemorrhage in pregnancy with acute aortic dissection. Early identification, early diagnosis, multidisciplinary collaborative therapy, and effective timing of vascular surgery and pregnancy termination determine the survival outcomes of the mother and foetus. Therefore, it is necessary to formulate diagnosis and treatment standards for pregnancy with acute aortic dissection in line with China’s national conditions. These standards can be used to guide clinical diagnosis and treatment.
